# Effect of Curdlan on the Structural Stability and Thermal Processing Properties of Mycelium-Based Gels Used in 4D-Printed Meat Analogs

**DOI:** 10.3390/gels12050453

**Published:** 2026-05-21

**Authors:** Xin Hu, Jingyu Wang, Haijin Tang, Xinlian Su, Lifang Zou, Baocai Xu

**Affiliations:** 1 School of Food and Biological Engineering, Hefei University of Technology, Hefei 230601, China; xhuxin@126.com (X.H.); haijintang0801@126.com (H.T.); suanmoqin@163.com (X.S.); zoulifang526@163.com (L.Z.); 2 Anhui Ecological Fermentation Engineering Research Center for Functional Fruit Beverage, Fuyang Normal University, Fuyang 236037, China; jimixy8072@yeah.net

**Keywords:** meat analogs, mycelium-based foods, food 3D/4D printing, curdlan, thermal processing, structural stability

## Abstract

This study investigated the effects of curdlan (CUR) on the structural stability and thermal processing properties of *Pleurotus eryngii* mycelium–soy protein isolate–cassava starch gels used as bio-ink scaffolds for 4D-printed meat analogs. Bio-inks containing different CUR concentrations (0–5%, *w*/*w*) were prepared, and their rheological properties, 3D printability, microstructure, and water distribution were systematically evaluated. The fermented meat analogs were then subjected to steaming and baking to assess cooking loss, dimensional shrinkage, and textural properties. The results showed that CUR significantly increased the yield stress and structural recovery of the bio-inks while maintaining high height retention (>87%), thereby providing a favorable scaffold for mycelial growth and subsequent product formation. During thermal processing, CUR effectively mitigated structural collapse, which may be attributed to its heat-induced thermally irreversible gelation and the formation of an internal supporting network that resisted matrix contraction and dehydration. In particular, the addition of 5% CUR reduced cooking loss from 12.83% to 7.35% during steaming and from 42.52% to 38.59% during baking, while reducing shrinkage to 9.29% and 18.00%, respectively. In addition, hardness, springiness, and chewiness were significantly improved after cooking. Overall, CUR functioned not only as a rheological modifier for extrusion printing but also as a heat-activated internal supporting network during cooking, owing to its thermally irreversible gelation.

## 1. Introduction

The rapid growth of the global population and the associated increase in meat consumption have imposed substantial environmental pressures and public health concerns, thereby intensifying the demand for sustainable alternative proteins [1]. Among the various alternative proteins, mycelium has emerged as a highly promising candidate [2]. Unlike plant proteins that require intensive high-temperature and high-shear extrusion processes, fungal mycelium inherently possesses a complex, three-dimensional branching network [3]. This structure more closely resembles the texture of animal muscle and is accompanied by an excellent nutritional profile rich in high-quality protein, dietary fiber, and bioactive compounds [4].

An extension of 3D printing technology, 4D printing incorporates the dimension of time, enabling printed constructs to undergo changes in shape or properties in response to external stimuli such as temperature, humidity, light, and enzymes [5,6,7,8]. Beyond these conventional physical and chemical triggers, recent studies have explored biological growth as a driving stimulus for 4D bioprinting [9]. It can be combined with various matrix materials to form composites with distinct functional properties [10]. The biological stimulus 4D printing can result in transformations that are not limited to morphology and structure but may also involve changes in mechanical properties, color, flavor release, and nutrient bioavailability [11,12]. This dynamic responsiveness offers new opportunities to optimize the structural design and sensory performance of food products and has therefore attracted increasing attention in the food sector [13].

**Figure 1:**
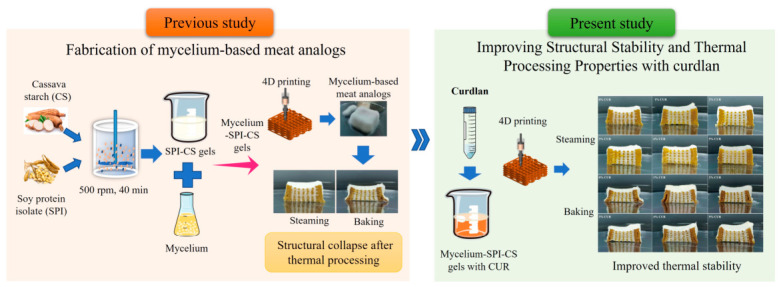
Schematic overview of the previous fabrication strategy and the present curdlan (CUR) reinforcement approach for improving the thermal processing stability of printed mycelium-based meat analogs.

In our previous study, we developed an integrated strategy combining 4D printing with solid-state fermentation to fabricate mycelium-based meat analogs using soy protein isolate (SPI), cassava starch (CS), and *Pleurotus eryngii* mycelium as the core materials. Specifically, a porous SPI-CS scaffold was first fabricated by 3D printing to guide the colonization and proliferation of *Pleurotus eryngii* mycelium within the pore structure, thereby forming a dense internal network (Figure 1). This process achieved biological growth stimuli-responsive 4D printing, which accomplished solid-state fermentation, while simultaneously enhancing the mechanical strength of the meat analogs [14]. However, these mycelium-based meat analogs remain susceptible to structural instability during high-temperature processing, which can lead to structural collapse, excessive cooking loss, severe shrinkage, and deterioration of textural properties such as chewiness and elasticity.

To mitigate this thermally induced deterioration, the incorporation of functional hydrocolloids is considered an effective strategy. Curdlan (CUR), a water-insoluble extracellular β-1,3-glucan produced by microorganisms, is particularly notable for its unique thermal gelation behavior [15]. When heated above 80 °C, CUR forms a highly cross-linked, thermally irreversible, and resilient gel network [16]. This high-temperature gelation characteristic is well-suited to the thermal processing conditions used for meat analogs. In addition, the elastic and firm texture of CUR gels may help mimic the bite and chewiness associated with animal connective tissue and fat [17]. We hypothesized that beyond improving the rheology of inks during printing, CUR would form a thermally irreversible supporting network during heating, thereby enhancing the structural stability of 4D-printed mycelium-based meat analogs. This dual-stage functionality distinguishes previous studies that mainly focused on hydrocolloid-assisted printability or CUR-reinforced gel texture.

Accordingly, this study aimed to investigate the effects of CUR on the structural stability and thermal processing properties of *Pleurotus eryngii* mycelium–SPI–CS gels used as bio-inks for 4D-printed meat analogs. Specifically, different concentrations of CUR (1–5%, *w*/*w*) were incorporated into an SPI–CS–mycelium composite bio-gel. The effects of CUR on ink rheology, printability, microstructure, and water distribution were systematically evaluated. In addition, the cooking loss, dimensional shrinkage, and texture profiles (e.g., hardness, springiness, and chewiness) of the fermented mycelium-based meat analogs under different thermal treatments (steaming and baking) were comprehensively analyzed. The findings are expected to provide both theoretical support and practical guidance for improving the thermal processing quality of novel mycelium-based meat analogs.

## 2. Results and Discussion

### 2.1. Rheological Properties of Bio-Inks

**Figure 2:**
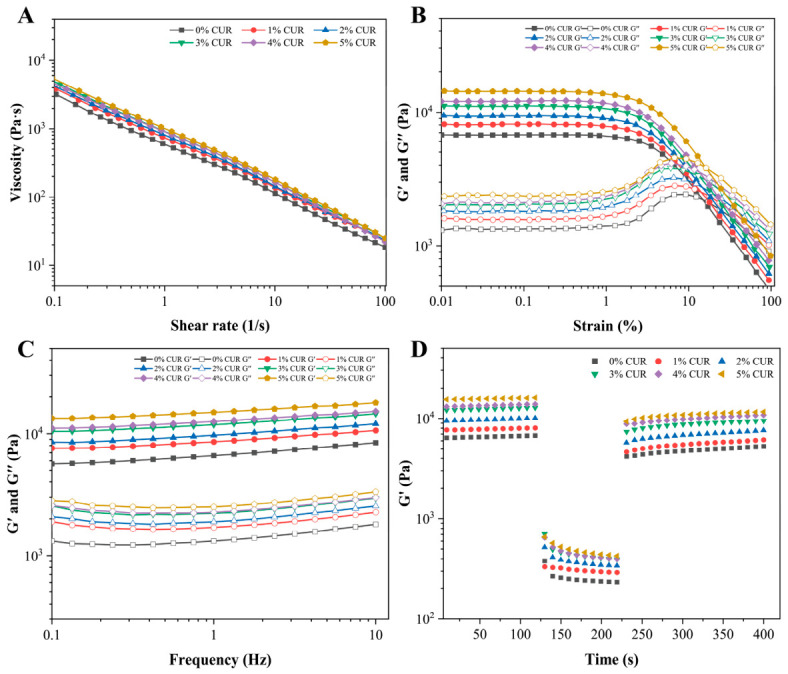
Rheological properties of mycelium inks with different curdlan (CUR) concentrations. (**A**) Shear viscosity; (**B**) Amplitude sweep; (**C**) Frequency sweep; (**D**) 3ITT.

The rheological properties of mycelial bio-inks are crucial for extrusion behavior, filament formation, and post-printing structural stability [18]. As shown in Figure 2A, the viscosity of all formulations decreased with increasing shear rate, indicating typical shear-thinning behavior [19]. At a given shear rate, the viscosity increased progressively with CUR concentration. Specifically, at 0.1 s^−1^, the viscosity of the 5% CUR formulation was 73.14% higher than that of the CUR-free control.

To quantitatively describe the flow behavior of the mycelium-based bio-inks, the viscosity data were fitted using the Power-law model [20], and the fitting parameters are summarized in Table 1. The model showed good fitting performance for all formulations, as indicated by the high *R*^2^ values. The flow behavior index *n* of all samples was lower than 1, confirming that the bio-inks exhibited typical pseudoplastic and shear-thinning behavior. This result is consistent with the viscosity curves shown in Figure 2A.

With increasing CUR concentration, the consistency coefficient *K* increased, indicating enhanced flow resistance and network strength of the composite ink system [21]. The increase in *K* may be associated with the thickening effect of CUR and the formation of a denser physical network within the SPI–CS–mycelium matrix. From the perspective of extrusion-based 3D printing, this behavior is beneficial because the ink can flow smoothly through the nozzle under high shear.

Figure 2B illustrates the results of the amplitude sweep tests. Within the low strain region (0.01–1%), both the storage modulus (G′) and loss modulus (G″) of all ink formulations remained relatively constant, indicating that the inks were in the linear viscoelastic region and that their internal structures were not disrupted under small deformation [22]. As the strain increased beyond the linear viscoelastic region, G′ decreased markedly, indicating the breakdown of the gel network under large deformation. In addition, the yield stress of the 5% CUR formulation increased by 60.22% compared with the control, indicating that CUR improved the resistance of the ink to flow and deformation. From the perspective of printing, a higher yield stress is beneficial for maintaining the height and geometry of the printed construct after deposition [23].

The frequency sweep results are shown in Figure 2C. In all samples, G′ remained higher than G″ over the entire frequency range, further confirming the formation of stable, elasticity-dominant gel-like networks. The increase in G′ with CUR concentration suggests that CUR contributed to the formation of a stronger polymeric network within the composite inks [24]. This enhanced elasticity is favorable for layer-by-layer deposition because it improves the self-supporting capacity of the printed filaments and reduces deformation under gravity [25].

The three-interval thixotropy test (3ITT) was used to simulate the structural breakdown and recovery of the inks during extrusion printing (Figure 2D). In the first interval, the inks maintained high G′ values under low strain, reflecting the stable structure before extrusion. During the second interval, a high strain was applied to simulate the strong shear force experienced by the ink inside the nozzle, resulting in a sharp decrease in G′ due to network disruption. When the strain was reduced in the third interval, G′ recovered rapidly and approached a stable value within 100 s. This rapid recovery indicates that the disrupted network could be rebuilt after extrusion, which is important for maintaining filament integrity and shape fidelity of the printed constructs [26].

### 2.2. 3D Printability and Structural Stability

**Figure 3:**
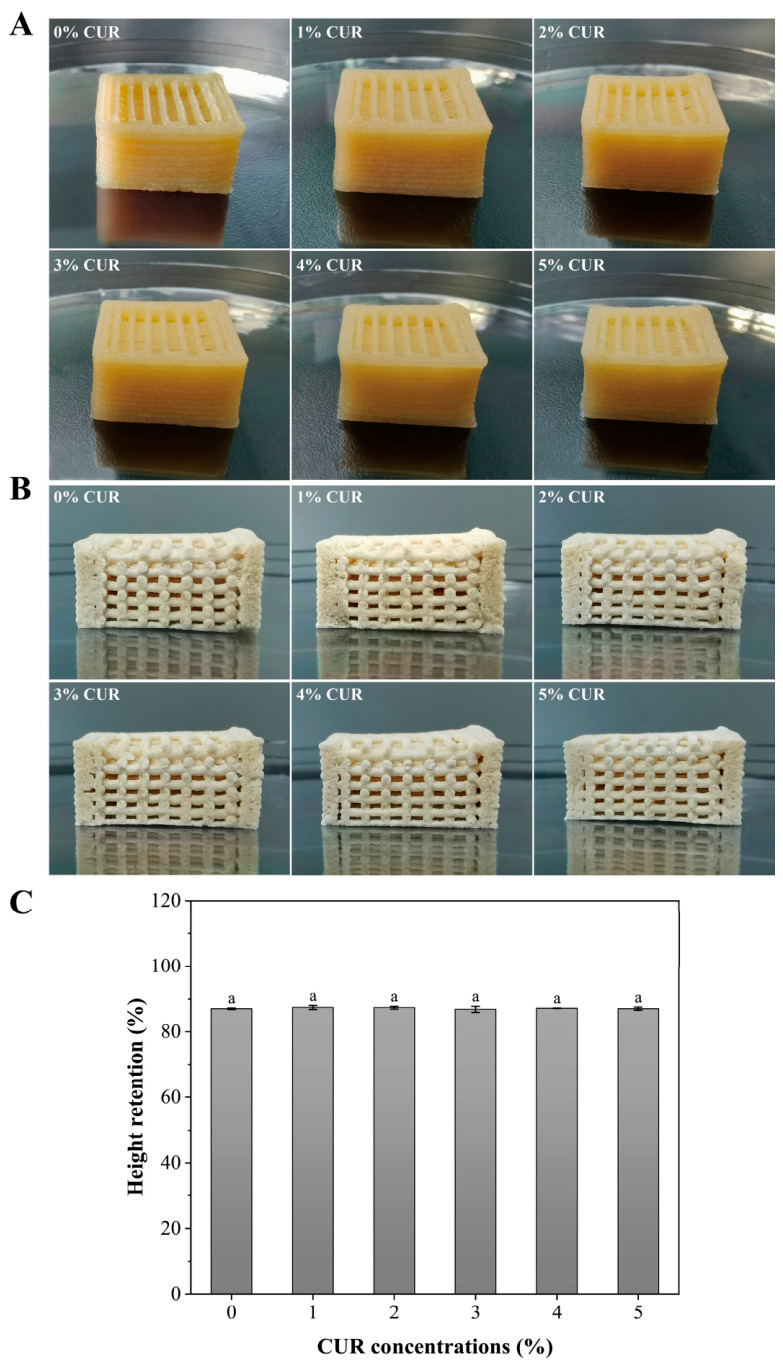
Printability of mycelium inks with different curdlan (CUR) concentrations. (**A**) Apparent morphology; (**B**) Internal structure; (**C**) Height retention. Note: Different lowercase letters indicate significant differences among groups.

The printability of a bio-ink is generally evaluated by its ability to be extruded into continuous filaments and to form a self-supporting structure that retains the designed geometry rapidly [27]. The macroscopic appearance and internal structures of the printed scaffolds with different CUR contents are shown in Figure 3. All formulations were successfully printed with continuous filaments. Moreover, the printed constructs remained morphologically stable with no apparent gravity-induced collapse. This favorable macroscopic fidelity is consistent with the higher yield stress and rapid structural recovery observed in the rheological analysis [28].

Beyond macroscopic appearance, the integrity of the internal structure is particularly important for the subsequent solid-state fermentation process [29]. Cross-sectional images of the freeze-dried printed constructs (Figure 3B) showed that all groups retained an intact grid-like internal architecture. The pores exhibited clear boundaries and a relatively uniform distribution, with no obvious interlayer fusion. Such an interconnected porous structure can provide sufficient space for the colonization and proliferation of *Pleurotus eryngii* mycelium while also facilitating oxygen and nutrient transport during fermentation [5]. Therefore, it offers an appropriate structural basis for in situ mycelial growth and the subsequent 4D transformation of the meat analogs. Vertical deformation is a key indicator of print stability because collapse under gravity reduces pore height and volume, promotes interlayer fusion, and hinders oxygen and nutrient transport [14]. Accordingly, height retention was used to evaluate preservation of the internal growth space. As shown in Figure 3C, all formulations maintained height retention above 87%, indicating good resistance to vertical collapse.

Overall, the incorporation of 1–5% CUR increased the viscoelastic moduli of the inks, thereby improving the structural stability and deformation resistance of the printed constructs without adversely affecting extrudability. These results indicate that CUR contributed to the stable fabrication of the target models. Similar rheological improvements have been reported in previous studies, in which the incorporation of functional additives reinforced non-covalent interactions within hydrogel-based inks and improved structural fidelity and post-extrusion shape retention [21,30].

### 2.3. Microstructure of the Bio-Inks

**Figure 4:**
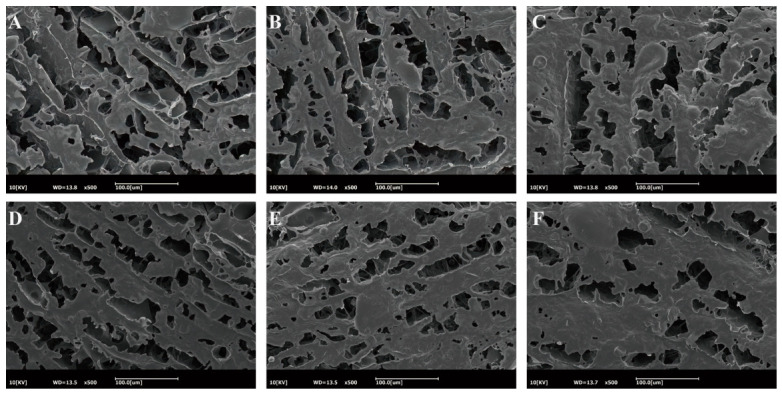
Microstructure of mycelium inks with different curdlan concentrations. ((**A**–**F**): 0%, 1%, 2%, 3%, 4%, and 5% curdlan, respectively).

Scanning electron microscopy (SEM) was used to characterize the microstructure of mycelium inks containing different levels of CUR. As shown in Figure 4, the CUR-free ink exhibited a relatively loose and porous network, with non-uniform pore sizes and numerous irregular voids. As the CUR content increased (Figure 4B–F), the microstructure underwent marked changes, evolving into a progressively denser network characterized by smaller pore sizes, a more uniform pore distribution, and a visibly thickened structural skeleton. This structural change may be attributed to the linear β-1,3-glucan chains of CUR, which can form a robust three-dimensional network [15]. Within the mycelium ink system, CUR acts as a structural filler, occupying voids within the SPI–CS–mycelium matrix and thereby densifying the composite network. The formation of this highly compact and uniform structure at higher CUR levels aligns well with the improved printability and structural stability of the constructs. Importantly, although the matrix becomes denser, it still retains an interconnected porous structure; this residual porosity is crucial for subsequent mycelial colonization, proliferation, and nutrient transport [31].

### 2.4. Water Distribution of the Fermented Meat Analogs

**Figure 5:**
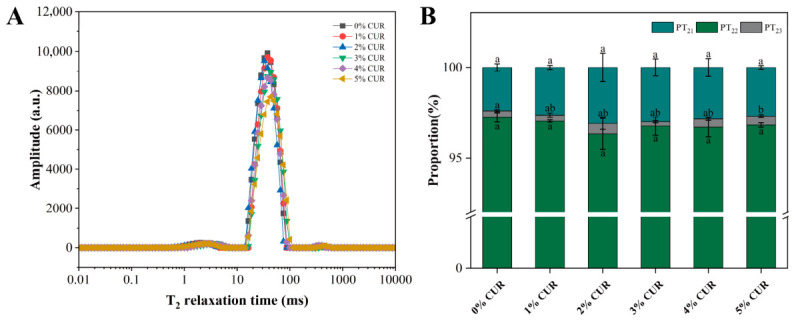
Water distribution of mycelium-based meat analogs with different curdlan (CUR) concentrations. (**A**) T_2_ relaxation time distribution spectra; (**B**) Relative peak area proportions. Note: Different lowercase letters indicate significant differences among groups.

To evaluate the effect of CUR addition on water status and distribution in the fermented mycelium-based meat analogs, samples from each group were analyzed by LF-NMR (Figure 5). According to the transverse relaxation time (T_2_) inversion results, water in the system was mainly present in three states: bound water (T_21_), immobilized water (T_22_), and free water (T_23_). Figure 5B illustrates the relative proportions of these three water populations (PT_21_, PT_22_, and PT_23_, respectively). Across all samples, immobilized water was the dominant water fraction (PT_22_ > 96%). Although slight variations were observed among formulations, the proportions of bound, immobilized, and free water did not differ significantly with increasing CUR concentration, indicating that the overall water distribution remained relatively stable after fermentation.

CUR contains numerous hydroxyl groups, which can promote hydration and water retention through hydrogen bonding [32]. Wu et al. [33] reported that, in myofibrillar protein emulsion gels, increasing CUR concentration gradually converted internal moisture into bound water. In the present study, the relatively stable water distribution may be related to the prolonged 10 d solid-state fermentation process. During fermentation, hydrolysis of the protein matrix and the extensive penetration of proliferating mycelia into the base matrix may have counterbalanced the changes in water distribution that would otherwise be induced by increasing CUR concentrations. As a result, the LF-NMR data showed no significant differences in water distribution among the different CUR formulations.

### 2.5. Cooking Loss and Shrinkage

**Figure 6:**
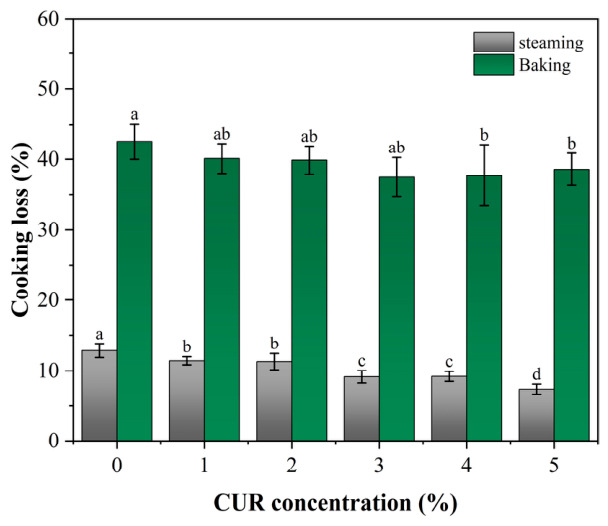
Cooking loss of mycelium-based meat analogs with different curdlan (CUR) concentrations. Note: Different lowercase letters indicate significant differences among groups.

Cooking loss reflects the extent of moisture loss during thermal processing and is closely related to a product’s yield, juiciness, and overall sensory quality [34]. As shown in Figure 6, cooking loss under both thermal treatments decreased significantly with increasing CUR concentration, and the values for baked samples were consistently higher than those for steamed samples. Under steaming conditions, the cooking loss of the CUR-free control was 12.83%, which gradually decreased to 7.35% as the CUR concentration increased to 5%. Under baking conditions, the cooking loss of the 0% CUR group reached 42.52%, whereas the 5% CUR formulation reduced this value to 38.59%. The higher cooking loss during baking was likely related to the high-temperature, low-humidity environment, which accelerates surface evaporation and promotes the migration and loss of internal water [35]. In contrast, the high-humidity environment during steaming suppresses moisture evaporation, thereby reducing the overall cooking loss [36].

It should be noted that the absence of significant differences in PT_21_, PT_22_, and PT_23_ does not necessarily indicate that all formulations would exhibit the same water retention behavior during heating. At elevated temperatures, CUR can form a thermally irreversible gel network, which may limit the water escape [37].

**Figure 7:**
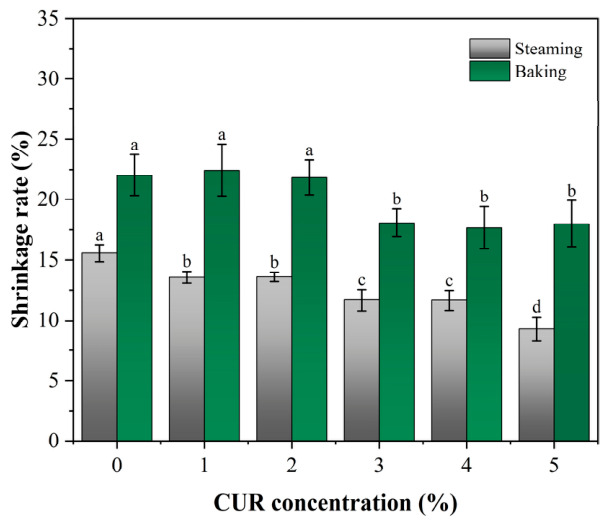
Shrinkage rate of mycelium-based meat analogs with different curdlan (CUR) concentrations after cooking. Note: Different lowercase letters indicate significant differences among groups.

Similarly, cooking shrinkage reflects the ability of a product to retain its shape after thermal processing and therefore directly affects product quality [34]. The shrinkage results of mycelium-based meat analogs with different CUR concentrations are shown in Figure 7. Under both steaming and baking conditions, shrinkage decreased significantly with increasing CUR concentration. In addition, the shrinkage values of baked samples were consistently higher than those of steamed samples. Under steaming conditions, the shrinkage of the CUR-free control was 15.55%, whereas it decreased to 9.29% in the 5% CUR group. Under baking conditions, which involved more severe dehydration, the shrinkage of the control group reached 22.02%, while the addition of 5% CUR reduced this value to 18.00%.

In general, high-temperature processing promotes protein denaturation and network contraction, usually accompanied by rapid moisture loss [38]. These changes can lead to densification and partial collapse of the gel matrix, thereby aggravating macroscopic shrinkage and deformation [36]. The reduced deformation observed in CUR-containing samples was likely related to the thermal gelation and structural reinforcement effects of CUR [37]. At temperatures above 80 °C, CUR undergoes further aggregation and network formation, which may help counteract the contractile forces associated with protein denaturation [39]. From a practical perspective, these reductions in cooking loss and shrinkage are important for the industrial application of 4D-printed mycelium-based meat analogs. This is relevant not only to product juiciness and consumer-perceived quality, but also to cost-efficiency during scale-up production [40].

### 2.6. Textural Properties

As shown in Table 2, CUR concentration significantly affected the textural properties of the thermally processed mycelium-based meat analogs. For all CUR formulations, the baked samples exhibited higher hardness and chewiness than the steamed samples. In the 5% CUR group, the hardness and chewiness values of the baked samples were 1.59-fold and 1.52-fold higher than those of the steamed samples, respectively. This difference was likely attributed to the high-temperature and low-humidity baking environment, which causes extensive moisture evaporation, leading to shrinkage and densification. Additionally, the higher temperature accelerates the conformational transition and intense aggregation of CUR, culminating in a tighter and more rigid three-dimensional network [41]. Simultaneously, the rapid loss of surface moisture induces the formation of a hard crust, which further enhances the overall textural strength.

Under the same thermal treatment, both hardness and chewiness increased significantly with increasing CUR concentration. After steaming, the hardness and chewiness of the 5% CUR group increased by 153.39% and 258.76%, respectively, compared with those of the CUR-free control. Similarly, after baking, these two parameters increased by 63.25% and 102.44%, respectively.

Springiness and cohesiveness also increased significantly with the increasing CUR concentration (*P* < 0.05). Under steaming conditions, the springiness and cohesiveness of the 5% CUR group increased by 12.91% and 25.41%, respectively, compared to the control. Under baking conditions, the corresponding increases were 7.44% and 15.06%, respectively. The increases in springiness and cohesiveness further indicate that CUR improved the elastic recovery and internal bonding strength of the thermal meat analogs [21]. Adhesiveness, which primarily characterizes the work required to detach the testing probe from the sample surface [42], did not differ significantly among formulations. One possible explanation is that the surfaces of the fermented samples were covered by a dense mycelial layer, which may have masked differences in the adhesiveness of the internal matrix.

### 2.7. Internal Structure After Cooking

**Figure 8:**
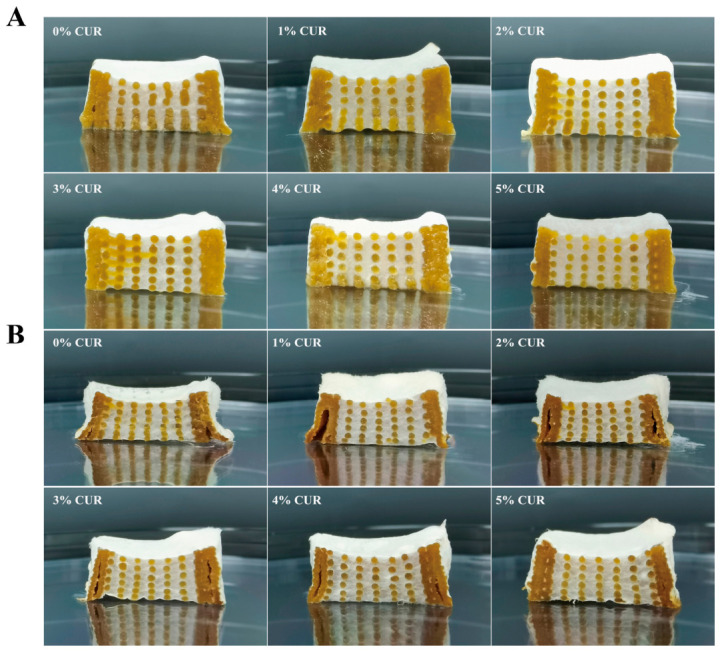
Internal structure of mycelium-based meat analogs with different curdlan (CUR) concentrations after thermal processing. (**A**) Steaming; (**B**) Baking.

To evaluate the effect of CUR addition on the structural stability of solid-state-fermented mycelium-based meat analogs during thermal processing, the cross-sectional structures of samples subjected to steaming and baking were examined (Figure 8). Steaming is a typical moist heat processing method characterized by high environmental humidity [43]. As shown in Figure 8A, the CUR-free sample exhibited pronounced lateral swelling and bottom sagging after steaming. This deformation was likely associated with severe softening of the internal matrix caused by the penetration of high-temperature steam. In contrast, when the CUR concentration reached 3% or higher, bottom softening and sagging were markedly reduced, and the samples retained a more regular geometry.

Baking, by contrast, is a dry-heat treatment in which high temperature accelerates moisture evaporation and generates greater shrinkage stress [35]. After baking, samples with no or low CUR showed obvious inward concave deformation along the sidewalls, suggesting that the SPI-CS matrix and mycelial network alone did not provide sufficient mechanical strength to maintain structural integrity under dehydration conditions (Figure 8B). As the CUR content increased, both the macroscopic shape fidelity and the integrity of the internal pore structure were progressively improved.

The improved structural stability was likely associated with the heat-induced gelation of CUR. These structural observations visually support the reductions in cooking loss and shrinkage and the improvements in textural properties, indicating that CUR helped preserve the internal architecture during heating.

## 3. Conclusions

This study demonstrated that CUR improved the structural stability of 4D-printed, solid-state-fermented mycelium-based meat analogs throughout printing, fermentation, and thermal processing. The addition of 1–5% CUR increased the yield stress and structural recovery of the bio-inks while maintaining high height retention (>87%) of printed scaffolds, thereby supporting mycelial growth. Among the tested formulations, 5% CUR showed the best overall performance. Compared with the CUR-free control, it reduced the cooking loss of the mycelium-based meat analogs from 12.83% to 7.35% during steaming and from 42.52% to 38.59% during baking. Concurrently, structural shrinkage decreased to 9.29% and 18.00%, respectively. Additionally, CUR also improved hardness, springiness, cohesiveness, and chewiness after cooking. In this system, CUR served a dual role by improving ink printability and reinforcing structural stability during heating. These findings provide a useful strategy for improving the thermal stability of 4D-printed mycelium-based meat analogs and highlight the advantage of CUR beyond simple viscosity enhancement. Although this study demonstrates the feasibility of the approach at the laboratory scale, several challenges remain for industrial implementation, including large-scale production, microbiological safety, and precise control of biomass development during incubation to ensure uniform product quality. Future studies should also evaluate sensory profiles, consumer acceptance, and the broader applicability of this approach to different protein materials, microbial strains, and processing systems.

## 4. Materials and Methods

### 4.1. Materials

Soy protein isolate (SPI) and cassava starch (CS) were purchased from commercial suppliers. Curdlan (CUR) powder was obtained from Shandong Tianzhi Lvye Biotechnology Co., Ltd. (Linyi, China). The *Pleurotus eryngii* strain was obtained from Shandong Yaosheng Biotechnology Co., Ltd. (Linyi, China). Potato dextrose broth and potato dextrose agar were purchased from Guangzhou Huankai Microbial Technology Co., Ltd. (Guangzhou, China).

### 4.2. Preparation of Mycelium Bio-Inks

The mycelium-based bio-inks were prepared according to the method reported by Hu et al. [14]. The base formulation was prepared with SPI (18% *w*/*w*) and CS (5% *w*/*w*) powders in sterile water, heated to 80 °C using a temperature-controlled steam oven (B9, CASDON, Shenzhen, China) for 30 min to induce gelation. To incorporate CUR, the powder was dispersed in sterile distilled water and stirred at 500 rpm for 10 min to prepare a 25% (*w*/*w*) suspension. The final bio-inks were then formulated by thoroughly mixing the SPI-CS gel with *Pleurotus eryngii* mycelium (6% *w*/*w*) and varying amounts of the CUR suspension to achieve final CUR concentrations of 0%, 1%, 2%, 3%, 4%, and 5% (*w*/*w*). The prepared bio-inks were stored at 4 °C before 3D printing and subsequent physicochemical analyses.

### 4.3. Rheological Measurements

The rheological properties of the mycelium-based bio-inks were measured using a rotational rheometer (Mars 40, Thermo Fisher Scientific, Waltham, MA, USA). The shear viscosity was recorded over a range of 0–100 s^−1^ to evaluate shear-thinning behavior, which is closely related to extrudability and shape retention in extrusion-based 3D printing. The flow behavior of the bio-inks was quantitatively described using the Power-law model [20]:

$$\tau =K{\dot{\gamma }}^{n}$$

where *τ* is the shear stress (Pa), *K* is the consistency coefficient (Pa·s*^n^*), $\dot{\gamma }$ is the shear rate (s^−1^), and *n* is the flow behavior index.

An amplitude sweep was performed over a strain range of 0.01–100% at 1 Hz to determine the linear viscoelastic region and yield stress [44]. A frequency sweep was then conducted over the range of 0.1–10 Hz to measure the G′ and G″ [45]. In addition, a 3ITT was performed to evaluate the structural recovery capacity of the inks after deposition. The 3ITT was conducted at 1 Hz and consisted of three sequential steps: (i) oscillation at 0.1% strain for 120 s; (ii) application of 100% strain for 100 s; and (iii) recovery at 0.1% strain for 180 s after high-strain disruption [46].

### 4.4. 3D Printing Process and Accuracy Evaluation

A food 3D printer (FOODBOT-EMCN, Hangzhou Shiyin Technology Co., Ltd., Hangzhou, China) was utilized to fabricate the scaffolds. The bio-inks were printed into cuboid models (20 × 20 × 10 mm^3^) using the following optimized parameters, which were selected based on our previous study [14]: infill density, 40%; nozzle diameter, 1 mm; printing speed, 15 mm/s; and extrusion temperature, 25 °C. Height retention was determined by comparing the measured heights of the printed samples with the theoretical height of the CAD model, according to the following equation:

$$Height\;retention\;(\%)=\frac{\left(1-\frac{\left|H_{Center}-H_{Design}\right|}{H_{Design}}\right)+\left(1-\frac{\left|H_{Edge}-H_{Design}\right|}{H_{Design}}\right)}{2}$$

where *H_Edge_*, *H_Center_*, and *H_Design_* represent the edge, center, and design height (mm), respectively.

### 4.5. Microstructure Analysis

The microstructures of the bio-inks with different CUR concentrations were observed using a scanning electron microscope (SEM, EM-30 Plus, COXEM, Daejeon, South Korea). Prior to observation, the ink samples were freeze-dried using a lyophilizer (SCIENTZ-50, Ningbo Scientz Biotechnology Co., Ltd., Ningbo, China) and sputter-coated with a thin layer of gold to improve conductivity [47].

### 4.6. Preparation of Fermented Mycelium-Based Meat Analogs

The 3D printed scaffolds were subjected to solid-state fermentation to obtain the final mycelium-based meat analogs. The printed samples were incubated under sterile conditions at 25 °C and 95% relative humidity for 10 d, allowing *Pleurotus eryngii* mycelium to fully proliferate and colonize the porous scaffold network [14].

### 4.7. Water Distribution Analysis

The water status and distribution of the fermented meat analogs were analyzed using a low-field nuclear magnetic resonance (LF-NMR) analyzer (NMI20-060H-I, Suzhou Niumag Analytical Instrument Corporation, Suzhou, China). The measurement parameters were set as follows: a proton resonance frequency of 20 MHz, echo time of 0.4 ms, wait time of 8000 ms, number of echoes of 15,000, and number of scans of 8. The transverse relaxation time (T_2_) was measured, and the relative peak area proportions (PT_2_) were calculated [19].

### 4.8. Thermal Processing and Cooking Properties

The fermented mycelium-based meat analogs were subjected to two thermal treatments: steaming and baking (B9, CASDON, Shenzhen, China). For steaming, the samples were heated in a steamer at 100 °C for 20 min. For baking, the samples were heated in a steam oven with the upper and lower heating elements set at 180 °C for 10 min.

Cooking loss: Cooking loss was determined by weighing the samples before and after thermal processing [36]. The results were calculated using the following equation:

Cooking loss (%) = [(W_0_ − W_1_)/W_0_] × 100

where W_0_ represents the initial weight before cooking, and W_1_ represents the weight after cooking.

Shrinkage rate: The shrinkage rate was defined as the percentage reduction in sample length after cooking relative to that of the raw fermented samples and was measured using a digital vernier caliper [36].

Internal structure observation: The steamed and baked samples were cut along the midline, and their internal cross-sections were photographed using a digital camera (Mate 40, Huawei Technologies Co., Ltd., Shenzhen, China) with an aperture of f/2.2 and a focal length of 2 mm. To ensure visual consistency, the photographs were taken immediately after cutting. The camera lens was positioned at a vertical distance of 10 cm from the sample surface, and all images were acquired under uniform ambient laboratory lighting.

### 4.9. Texture Profile Analysis

The textural properties of the raw and thermally processed meat analogs were measured using a texture analyzer (TA-XT Plus, Stable Micro Systems, Godalming, UK) equipped with a P/36R probe. The samples were compressed to 50% of their original height at a test speed of 1.0 mm/s with a trigger force of 5 g. Textural parameters, including hardness, adhesiveness, springiness, cohesiveness, and chewiness, were obtained [48].

### 4.10. Statistical Analysis

All experiments were performed at least in triplicate, and the data are presented as mean ± standard deviation (SD). Statistical analysis was conducted using analysis of variance (ANOVA), and significant differences between groups were determined by Duncan’s multiple range test at a confidence level of *p* < 0.05.

## Figures and Tables

**Table 1:** Fitting parameters of the Power-law model for mycelium-based bio-inks with different curdlan (CUR) concentrations.

CUR Concentration	*K* (Pa·s*^n^*)	*n*	*R* ^2^
0% CUR	640.233 ± 22.01 ^e^	0.2313 ± 0.0110 ^b^	0.9938 ± 0.0017
1% CUR	722.53 ± 26.22 ^d^	0.2500 ± 0.0039 ^a^	0.9958 ± 0.0013
2% CUR	802.13 ± 0.902 ^c^	0.2459 ± 0.0042 ^a^	0.9960 ± 0.0003
3% CUR	900.97 ± 13.94 ^b^	0.2307 ± 0.0042 ^b^	0.9918 ± 0.0024
4% CUR	915.23 ± 14.88 ^b^	0.2207 ± 0.0082 ^bc^	0.9891 ± 0.0038
5% CUR	1018.67 ± 15.28 ^a^	0.2157 ± 0.0068 ^c^	0.9894 ± 0.0027

Note: Data are presented as mean ± standard deviation, n = 3. *K*, consistency coefficient; *n*, flow behavior index; *R*^2^, coefficient of determination. Different lowercase letters within the same column indicate significant differences among groups.

**Table 2:** Effects of curdlan (CUR) concentration and thermal processing conditions on the textural properties of mycelium-based meat analogs.

	Hardness (g)		Adhesiveness (g.s)		Springiness		Cohesiveness		Chewiness	
	Steam	Bake	Steam	Bake	Steam	Bake	Steam	Bake	Steam	Bake
0% CUR	314.51 ± 3.63 ^f^	776.94 ± 32.05 ^d^	−40.61 ± 3.44 ^a^	−22.31 ± 2.27 ^a^	0.767 ± 0.005 ^f^	0.847 ± 0.024 ^b^	0.724 ± 0.007 ^d^	0.717 ± 0.022 ^c^	174.76 ± 3.32 ^f^	471.05 ± 4.07 ^e^
1% CUR	366.89 ± 5.38 ^e^	813.76 ± 20.13 ^d^	−39.65 ± 3.30 ^a^	−22.98 ± 0.78 ^a^	0.780 ± 0.004 ^e^	0.857 ± 0.013 ^b^	0.743 ± 0.014 ^d^	0.725 ± 0.009 ^c^	212.59 ± 3.64 ^e^	505.49 ± 10.90 ^de^
2% CUR	465.32 ± 4.13 ^d^	884.96 ± 73.82 ^cd^	−40.18 ± 8.03 ^a^	−23.44 ± 2.59 ^a^	0.793 ± 0.007 ^d^	0.866 ± 0.018 ^b^	0.782 ± 0.006 ^c^	0.778 ± 0.013 ^b^	288.82 ± 12.83 ^d^	597.50 ± 72.67 ^cd^
3% CUR	547.50 ± 13.86 ^c^	997.27 ± 97.51 ^bc^	−40.77 ± 1.25 ^a^	−22.61 ± 0.76 ^a^	0.811 ± 0.003 ^c^	0.869 ± 0.026 ^ab^	0.809 ± 0.010 ^c^	0.807 ± 0.006 ^a^	359.23 ± 12.68 ^c^	699.46 ± 66.66 ^bc^
4% CUR	633.39 ± 7.81 ^b^	1066.15 ± 45.24 ^b^	−43.44 ± 1.86 ^a^	−22.56 ± 1.01 ^a^	0.830 ± 0.003 ^b^	0.885 ± 0.021 ^ab^	0.841 ± 0.036 ^b^	0.816 ± 0.010 ^a^	441.84 ± 15.97 ^b^	770.18 ± 18.32 ^b^
5% CUR	796.94 ± 20.52 ^a^	1268.35 ± 84.70 ^a^	−41.96 ± 2.15 ^a^	−22.44 ± 1.47 ^a^	0.866 ± 0.001 ^a^	0.910 ± 0.029 ^a^	0.908 ± 0.004 ^a^	0.825 ± 0.006 ^a^	626.97 ± 18.74 ^a^	953.58 ± 100.43 ^a^

Note: Data are presented as mean ± standard deviation, n = 3. Different lowercase letters within the same column indicate significant differences among groups.

## Data Availability

Data will be made available on request.
